# The Sigma Class Glutathione Transferase from the Liver Fluke *Fasciola hepatica*

**DOI:** 10.1371/journal.pntd.0001666

**Published:** 2012-05-29

**Authors:** E. James LaCourse, Samirah Perally, Russell M. Morphew, Joseph V. Moxon, Mark Prescott, David J. Dowling, Sandra M. O'Neill, Anja Kipar, Udo Hetzel, Elizabeth Hoey, Rafael Zafra, Leandro Buffoni, José Pérez Arévalo, Peter M. Brophy

**Affiliations:** 1 Institute of Biological, Environmental and Rural Sciences, Aberystwyth University, Aberystwyth, Wales, United Kingdom; 2 Molecular and Biochemical Parasitology Group, Liverpool School of Tropical Medicine, Liverpool, England, United Kingdom; 3 School of Biological Sciences, University of Liverpool, Liverpool, England, United Kingdom; 4 Faculty of Science and Health, Dublin City University, Dublin, Ireland; 5 Faculty of Veterinary Science, University of Liverpool, Liverpool, England, United Kingdom; 6 School of Biological Sciences, Queen's University of Belfast, Belfast, Northern Ireland, United Kingdom; 7 School of Veterinary Medicine, University of Córdoba, Córdoba, Spain; University of Queensland, Australia

## Abstract

**Background:**

Liver fluke infection of livestock causes economic losses of over US$ 3 billion worldwide per annum. The disease is increasing in livestock worldwide and is a re-emerging human disease. There are currently no commercial vaccines, and only one drug with significant efficacy against adult worms and juveniles. A liver fluke vaccine is deemed essential as short-lived chemotherapy, which is prone to resistance, is an unsustainable option in both developed and developing countries. Protein superfamilies have provided a number of leading liver fluke vaccine candidates. A new form of glutathione transferase (GST) family, Sigma class GST, closely related to a leading Schistosome vaccine candidate (Sm28), has previously been revealed by proteomics in the liver fluke but not functionally characterised.

**Methodology/Principal Findings:**

In this manuscript we show that a purified recombinant form of the *F. hepatica* Sigma class GST possesses prostaglandin synthase activity and influences activity of host immune cells. Immunocytochemistry and western blotting have shown the protein is present near the surface of the fluke and expressed in eggs and newly excysted juveniles, and present in the excretory/secretory fraction of adults. We have assessed the potential to use *F. hepatica* Sigma class GST as a vaccine in a goat-based vaccine trial. No significant reduction of worm burden was found but we show significant reduction in the pathology normally associated with liver fluke infection.

**Conclusions/Significance:**

We have shown that *F. hepatica* Sigma class GST has likely multi-functional roles in the host-parasite interaction from general detoxification and bile acid sequestration to PGD synthase activity.

## Author Summary

Combating neglected parasitic diseases is of paramount importance to improve the health of human populations and/or their domestic animals. Uncovering key roles in host-parasite interactions may support the vaccine potential portfolio of a parasite protein. *Fasciola hepatica* causes global disease in humans and their livestock but no commercial vaccines are available. Members of the Sigma class glutathione transferase (GST) family have long been highlighted as vaccine candidates towards parasitic flatworms. To this end, a Sigma class GST is currently undergoing phase II clinical trials to protect against infection from the schistosomes. In this study we characterise the protein from *F. hepatica* following four work pathways that 1) confirm its designation as a Sigma class GST using substrate profiling, 2) assess prostaglandin synthase activity and its effect on host immune cells, 3) localise the Sigma GST within adult fluke and between ontogenic stages and 4) measure its potential as a vaccine candidate. The work presented here shows *F. hepatica* Sigma class GST to have key host-parasite roles and we suggest, warrants further investigation for inclusion into vaccine formulations.

## Introduction

The liver flukes, *Fasciola hepatica* and *Fasciola gigantica* are the causative agents of fasciolosis, a foodborne zoonotic disease affecting grazing animals and humans worldwide [1]. Liver fluke causes economic losses of over US$ 3 billion worldwide per annum to livestock via mortality, reduction in host fecundity, susceptibility to other infections, decrease in meat, milk and wool production and condemnation of livers [1]. The disease is increasing in livestock worldwide with contributing factors such as climate change (warmer winters and wetter summers supporting larger intermediate mud snail host populations); fragmented disease management (only treating sheep not cattle and limiting veterinary interaction); encouragement of wet-lands; livestock movement; and/or failure/resistance of chemical control treatments in the absence of commercial vaccines [1], [2]. Fasciolosis is also a re-emerging human disease with estimates of between 2.4 and 17 million people infected worldwide [3]. In response, the World Health Organisation have added fasciolosis to the preventative chemotherapy concept [4].

There are currently no commercial vaccines and triclabendazole (TCBZ) is the most important fasciolicide, as the only drug with significant efficacy against adult worms and juveniles [5]. Evidence from developed countries where TCBZ has been used widely exposes the reliance on this drug as an Achilles heel of liver fluke chemotherapeutic control, with well-established evidence of drug-resistance [5]. Therefore, TCBZ does not offer a long-term sustainable option for livestock farmers worldwide. The need for a liver fluke vaccine is further underscored by the fact that the costs associated with anthelmintic intervention for fluke control make short-lived chemotherapy an unsustainable option in developing countries. Protein superfamily studies in liver fluke have provided a number of leading vaccine candidates. High quality one-gene based vaccine discovery research has identified several vaccine candidates from protein superfamilies that provide significant, but often variable protection rates in challenge animal trials against liver fluke.

For example, Mu class Glutathione transferase (GSTs) have been widely investigated as vaccine candidates for fasciolosis [6]–[9]. The Mu class GSTs have established roles in general Phase II detoxification of xenobiotic and endogenously derived toxins in *F. hepatica* within the host bile environment [10]. The general detoxification role is supported by GSTs contributing to 4% of the total soluble protein in *F. hepatica*, with a widespread tissue distribution. Proteomics and EST sequencing approaches have now delineated what members of the GST family are expressed in *F. hepatica* and two new classes of GST, Sigma and Omega, have been uncovered [11]. In the related trematode, *Schistosoma mansoni*, the Sigma class GST (Sm28) has generally shown more robust protection in vaccine trials against schistosome infection [12], than the *F. hepatica* Mu GSTs against *F. hepatica* infection.

Sigma class GSTs, unlike Mu Class GSTs, have been characterized as GSH-dependent hematopoietic prostaglandin synthases responsible for the production of prostaglandins in both mammals and parasitic worms [13]–[18]. Prostaglandins have been extensively studied in mammals and are shown to be involved in a range of physiological and pathological responses [19]–[23]. Parasite-produced prostaglandins may be involved in parasite development and reproduction as well as the modulation of host immunity, allergy and inflammation during establishment and maintenance of a host infection [16], [24]–[28]. The host protection success of Sigma GST based vaccinations in schistosomiasis may therefore be related to neutralising specific functions in host-parasite interplay, such as prostaglandin synthase activity.

In this manuscript we follow four work pathways to functionally characterise the newly identified Sigma GST from *F. hepatica*. 1) We confirm its designation as a Sigma class GST using substrate profiling, 2) we assess prostaglandin synthase activity and its effect on host immune cells, 3) we localise the Sigma GST within adult fluke and between ontogenic stages and 4) assess its potential as a vaccine candidate.

## Materials and Methods

### Sequence analysis

GST proteins representative of recognised GST superfamily classes were obtained from European Bioinformatics Institute Interpro database (http://www.ebi.ac.uk/interpro/), and from non-redundant databases at NCBI (http://www.ncbi.nlm.nih.gov/). A mammalian and a helminth or invertebrate GST sequence were selected for each GST class where available. Sequences were aligned via ClustalW program [29] in BioEdit Sequence Alignment Editor Version 7.0.5.2. [30] and sequence identity matrices produced from multiple alignments. Phylogenetic bootstrap neighbour-joining trees were produced as PHYLIP output files in ClustalX Version 1.83 [31] according to the neighbour-joining method of Saitou and Nei [32]. ClustalX default settings for alignments were accepted using the GONNET protein weight matrices with PHYLIP tree format files viewed within TREEVIEW [33].

### Recombinant *Fasciola hepatica* glutathione transferase Sigma class (rFhGST-S1) production

Full-length cDNA for FhGST-S1 was available in the form of an expressed sequence tag (EST) clone Fhep24h03, details of which can be obtained from the previously published Sigma class GST [11] and is identical to the submitted GenBank accession No. DQ974116.1 (NCBI http://www.ncbi.nlm.nih.gov/).

FhGST-S1 was amplified via PCR using the following primer pair: rFhGST-S1 forward primer, 5′ GGAATTC***CATATG***GACAAACAGCATTTCAAGTT 3′;rFhGST-S1 reverse primer, 5′ ATAAGAAT***GCGGCCGC***CTAGAATGGAGTTTTTGCACGTTTTTT 3′. Restriction enzyme sites (in bold type and underlined) for *Nde*I (forward primer) and *Not*I (reverse primer) were included so that the entire ORF could be directionally cloned into the pET23a (Novagen) vector. Recombinant protein was produced in *Escherichia coli* BL21(DE3) cells (Novagen).

### Protein purification of rFhGST-S1 and native *F. hepatica* GSTs

rFhGST-S1 protein was purified according to the glutathione affinity chromatography method of Simons and Vander Jagt [34] from transformed *E. coli* cytosol following protein expression. Native GSTs were purified from *F. hepatica* soluble cytosolic supernatants as previously described [11]. Purity of rFhGST-S1 was assessed by electrospray ionisation (ESI) mass spectrometry, sodium dodecyl sulphate polyacrylamide gel electrophoresis (SDS-PAGE) and 2DE according to LaCourse *et al.* [35].

### Substrate profiling of Sigma GST

A range of model and natural substrates (see Table 1 for details) were used to profile the Sigma GST. A number of ligands were also assessed for their ability to inhibit GST activity with 1-chloro-2, 4-dinitrobenzene (CDNB) as the second substrate [36]. Values were reported as the concentration of inhibitor required to bring GST specific activity to 50% of its original activity (IC50). At least six different inhibitor concentrations were used in each IC50 determination in triplicate. Inhibitors were pre-incubated for 5 minutes prior to starting reactions. IC50 values were estimated graphically [37].

**Table 1:** Substrate specificities of rFhGST-S1.

SUBSTRATE CLASS	SUBSTRATE	[Substrate] (mM)	[GSH] (mM)	100 mM KHPO_4_ (pH)	Temp. (°C)	l Max (nm)	e (mM^−1^cm^−1^)	rFhGST-S1	Sm28GST**	Assay Ref.
								Specific Activity (nmol min^−1^mg^−1^)	Specific Activity (nmol min^−1^mg^−1^)	
**MODEL SUBSTRATES**	1-Chloro-2,4-dinitrobenzene (CDNB)	1	1	6.5	25	340	9.6	4736±292	7269±218	[36]
	1,2-Dichloro-4-nitrobenzene (DCNB)	1	5	7.5	25	345	9.6	ND<5	ND<5	[36]
	Ethacrynic Acid	0.08	1	6.5	25	270	5	898±204	1580±97	[70]
**REACTIVE ALDEHYDES**	4-hydroxynonenal	0.1	0.5	6.5	30	224	13.75	645±129	287±17	[68]
	Trans-2-nonenal	0.023	1	6.5	25	225	−19.2	333±43	447±6	[36]
	Trans, trans-2,4-decadienal	0.023	1	6.5	25	280	−29.7	51±0.3	221	[36]
**LIPID PEROXIDES**	Cumene hydroperoxide	1.2	1	7	25	340	6.22	7081±1009	162±7	[71]
		0.25	1	7	25	340	6.22	2209±122	-	[71]
	t-butyl hydroperoxide	0.25	1	7	25	340	6.22	193±1.8	ND<10	[69]
		2.5	1	7	25	340	6.22	1827±198	-	[69]
	Linoleic Acid	0.25	1	7	25	340	6.22	430±69	-	[51]
		0.05	1	7	30	340	6.22	-	ND<10	[51]

Recombinant FhGST-S1 shows activity towards a broad range of model and natural GST substrates with a similar enzymatic profile to the Schistosomiasis vaccine trialist (Sm28GST - P09792). rFhGST-S1 also displays high glutathione-dependent lipid peroxidase activity compared to both Sm28GST and Sj26GST (Q26513) [47]. Reasonably high GSH-dependent lipid peroxidase activity has also been seen in a ‘weak affinity’ fraction following chromatofocusing of GSH transferase activity that failed to bind GSH-sepharose [10]. ND – Not determined.

**: Data taken from Walker *et al.* [47].

Prostaglandin synthase activity was assessed via an adapted method based upon those of Sommer *et al.* [26] and Meyer *et al.* [16], [38], with extraction modifications based upon Schmidt *et al.* [39]. In brief, reactions were performed in glass vials in 2 mM sodium phosphate buffer, pH 7.4, containing 10 mM glutathione, 50 mM NaCl, 0.5 mM tryptophan, 1 µM hematin, 1 U COX-1 enzyme, 100 µM arachidonic acid (All Sigma, UK. COX-1 [C0733]) and rFhGST-S1 at final concentration ranges of 0.1–100.0 µg/ml. Negative control reactions lacking either GST or COX-1 were also prepared. Reactions were incubated for 5–10 min in a water bath at 37°C. This was followed by 4 minutes incubation at 25°C in a shaking water bath. Prostaglandins were extracted by adding 860 µL of ice-cold ethyl acetate. Reactions were vortexed for 30 s then centrifuged briefly at 10,000× *g* at 4°C for 2 min. The upper ethyl acetate layer was retained and solvent was evaporated under a nitrogen stream at 45°C. The remaining residue was reconstituted in 50 µl of methanol/water/fomic acid (25∶75∶0,1) mix at pH 2.8 and stored at −80°C until ready for mass spectrometry analysis. Standards of prostaglandins D2, E2 and F2α (Cayman, Ltd) were also prepared in methanol/water/formic acid mix for analysis.

### Prostaglandin detection

The nano LC-MS analyses were performed using a Waters Q-Tof micro mass spectrometer (Waters) coupled to a LC-Packings Ultimate nano LC system (Dionex). The pre-column used was a LC Packings C18 PepMap 100 and the nano LC column used was a LC Packings 15 cm PepMap 100 C18 (both Dionex). Samples were loaded on the pre-column with mobile phase A (25% methanol with 0.1% formic acid added). Loading flow rate was 0.03 ml/min for 6 min. The samples were eluted on to the nano LC column using mobile phases B (60% acetonitrile) and C (100% methanol). A typical gradient profile was 100% B to 100% C in 10 min (flow rate of 0.2 µl/min) with the column held at 100% C for 1 hour. The mass spectrometer was operated in the negative ion nano electrospray mode with a source temperature of 80°C and capillary voltage 2.8 kV. The scan range was 40 to 400 Da for 1.5 s.

### Liver fluke extract and excretory/secretory (ES) product preparation

*F. hepatica* adults were collected, cultured *in vitro* for 4 h and the ES products collected and prepared as previously described [40]. Newly excysted juveniles (NEJ) were excysted from metacercariea *in vitro* and cultured in *Fasciola* saline for 4 h post excystment as previously described [41]. *F. hepatica* (adult and NEJ) soluble fractions were obtained by homogenisation of frozen fluke at 4°C in a glass grinder in lysis buffer (20 mM KHPO_4_, pH 7.0, 0.1% Triton-X100 and a cocktail of protease inhibitors [Roche, Complete-Mini, EDTA-free]). Homogenates were centrifuged at 100,000× *g* for 1 h at 4°C. Supernatants were considered as the soluble cytosolic fraction. Cytosolic protein extracts were treated and resolved by 2DE as described previously [11]. *F. hepatica* eggs were isolated, cultured and protein extracted as previously described [42].

### Western blotting

Recombinant *F. hepatica* Sigma GST (rFhGST-S1), and native *F. hepatica* S-hexylGSH-affinity purified GST samples (and human/rat recombinant PGD-synthase) were subjected to standard SDS-PAGE and 2DE, electro-transferred to membranes [43], [44] and western blotted with a polyclonal antibody (1∶20,000 dilution) raised in rabbits to the recombinant *F. hepatica* Sigma GST by Lampire Biological Laboratories, USA. Membranes were also probed with Mu class GST antibody (represented by the anti-*Schistosoma japonicum* GST26 Mu class antibody [1∶1,000 dilution] and an anti-rat PGD-synthase antibody [1∶1,000 dilution], Pharmacia-Biotech 27-4577). *F. hepatica* eggs, NEJs (somatic and ES preparations) and adults (somatic and ES preparations) were subjected to SDS-PAGE and also electro-transferred as described above and probed with the polyclonal antibody raised in rabbits to the recombinant *F. hepatica* Sigma GST. All western blots were developed as described previously [11].

### Immunolocalisation studies

*F. hepatica* Sigma class GST (FhGST-S1) was detected by immunohistology in tissue sections of whole adult *F. hepatica* extracted from bile ducts of sheep liver and also *in situ* from sections of liver.

Staining for FhGST-S1 was performed on formalin-fixed and paraffin-embedded tissue sections according to the method described previously [45]. Sections were washed in Tris-buffered saline (TBS; 0.1 M Tris-HCl with 0.9% NaCl [pH 7.2]), treated with 0.05% (w/v) protease (type XXIV, bacterial: Sigma) in TBS for 5 min at 37°C for antigen retrieval, before three further 5 min washes in ice-cold TBS. Following TBS washes, sections were incubated for 10 min in 50% (v/v) swine serum in TBS followed by incubation for 15–18 h at 4°C in rFhGST-S1 polyclonal antibody (diluted at 1∶500 in 20% swine serum in TBS). Sections were again washed in TBS before further incubation at ambient temperature (approximately 20°C+/−3°C) with anti-rabbit peroxidise anti-peroxidase (PAP; diluted at 1∶100 in 20% swine serum in TBS). Following washes with TBS, sections were incubated, with stirring, for 10 min, with 3,3-diaminobenzidine tetrahydrochloride (DAB; Fluka, Buchs, Switzerland) with 0.01% v/v hydrogen peroxide in 0.1 M imidazole buffer pH 7.1, before counterstaining with Papanicolaou's hematoxylin for 30 s. Sections were then rinsed, dehydrated in alcohol, cleared in xylene, and mounted. Consecutive sections from each tissue were used as negative controls in which the rFhGST-S1 polyclonal antibody was replaced by TBS.

### Induction of prostaglandin from dendritic cells

#### Animals

C57BL/6 mice were purchased from Harlan Ltd (UK) and TLR4KO (on a C57BL/6 background) bone marrow cells were a gift from Professor Padraic Fallon (Trinity College Dublin, Ireland). All mice were maintained according to the Irish Department of Children and Health.

#### Cell culturing and cytokine analysis

Bone marrow-derived immature dendritic cells (DCs) were prepared by culturing bone marrow cells isolated from the femurs and tibia of C57BL/6j and TLR4^−/−^ mice in complete RPMI 1640 (cRPMI; 5% [v/v] heat inactivated Fetal Calf Serum [FCS] [30 mins at 60°C], 100 U/ml penicillin, 100 µg/ml streptomycin, 2 mM L-glutamine and 50 µM 2-mercaptoethanol) with recombinant mouse GM-CSF (20 ng/ml; R&D Systems), at 37°C. On days 3 and 6 of culture, fresh medium with GM-CSF (20 ng/ml) was added to the cells. On day 8, cells were harvested, counted and stained with CD11c (Caltag Laboratories) for analysis by flow cytometry to determine purity (>90%). J774 cells and RAW264.7, murine macrophage cell lines were cultured in cRPMI 1640 medium containing 10% (v/v) FCS. All cells were used to conduct experiments when they reached ∼90% confluence.

For all experiments, cells were seeded into 24-well plates (Nunc) at 10^6^/ml in complete RPMI 1640 except for DCs where GM-CSF (5 ng/ml) was also added. Cells were treated with medium only, rFhGST-S1 (10 µg) or LPS (Alexa; 100 ng/ml) for 18 h. Levels of total prostaglandin (PG), prostaglandin E2 (PGE2) and prostaglandin D2 (PGD2) were measured using the Cayman competitive EIA. The values were calculated using free data analysis software available at www.caymanchem.com/analysis/eia. Data are presented as the mean ± SEM following subtraction of medium controls and are representative of two separate experiments. Prior to experimentation, rFhGST-S1 was assessed for endotoxin (i.e. LPS) contamination using the Pyrogene endotoxin detection system (Cambrex).

### Vaccination Strategy

#### Experimental design

Nineteen 5-month old, Malagueña breed goats were used for a vaccine trial. The animals were free of parasitic and infectious diseases as indicated by fecal analysis and absence of clinical signs. Group 1 (n = 10) were immunized with two subcutaneous injections of 100 µg of rFhGST-S1 in 1 ml of Quil A in 1 ml of PBS each injection separated by 4 weeks. Group 2 (n = 9) served as an infected control and was immunized at the same time with 1 ml of Quil A in 1 ml of PBS. Twelve weeks after the first immunization, animals were orally infected with 100 *F. hepatica* metacercariae of bovine origin. Three animals from each group were killed at 7, 8 and 9 days post-infection to study hepatic changes and host response during the early stages post-infection; the remaining animals (7 and 6 goats per group) were killed 15 weeks after infection to study fluke burdens, fecal egg counts and hepatic lesions. All goats were sacrificed by intravenous injection of thiobarbital. The experiment was approved by the Bioethical Committee of the University of Cordoba (N. 7119) and it was carried out according to European (86/609/CEE) and Spanish (RD 223/1988) directives for animal experimentation.

#### Fluke burdens and morphometrics

At necropsy, gallbladders and bile ducts were opened and flukes recovered. Llivers were cut (∼1 cm pieces) and washed in hot water to collect the remaining flukes. Flukes were counted, measured and weighed.

#### Fecal egg counts

Sedimentation techniques at 12 and 13 weeks after infection using four grams of feces were conducted to give eggs per gram (EPG).

#### Pathological assessment

At necropsy livers were photographed by the visceral and diaphragmatic aspects for gross pathology evaluation as described previously [46]. Gross hepatic lesions were scored as: absent [−]; mild [+] (less than 10% of hepatic surface affected); moderate [++] (10–25% of hepatic surface affected); severe [+++] (25–50% of hepatic surface affected) and very severe [++++] (more than 50% of hepatic surface affected). Tissue samples were collected from the left (6 samples) and right (2 samples) hepatic lobes, fixed in 10% buffered formalin and embedded in paraffin wax. Tissue sections (4 µm) were stained with haematoxylin and eosin (HE) for the histopathological study.

#### Specific IgG response

Specific IgG anti-rFhGST-S1were measured using the ELISA method as described previously [46]. A total of 10 µg/ml of rFhGST-S1 was used to coat microtitre plates, 100 µl/well of goat sera diluted in blocking buffer and rabbit anti-goat IgG peroxidase conjugated (whole molecule - Sigma) diluted in blocking buffer at 1∶10000. Serum pools were from ten experimentally infected goats and ten uninfected goats as positive and negative controls, respectively. All samples were analysed in duplicate. Results were expressed as antibody titre (Log10).

## Results

### Expression, purification and characterisation of rFhGST-S1

Aligning Sigma class GSTs of trematodes shows the extent of identity and similarity across this class of GSTs (Figure S1). An amino acid sequence comparison of FhGST-S1 with other trematode GSTs places FhGST-S1 into the Sigma class of GSTs, with identities averaging approximately 45%. Comparison with the most closely matching mammalian GSTs shows sequence identities averaging only approximately 28% (Table S1). Despite phylogenetic neighbour-joining trees place mammalian and trematode GSTs within the same broad Sigma class (Figure S1) there remains a distinct separation of the trematode and mammalian clusters.

**Figure 1:**
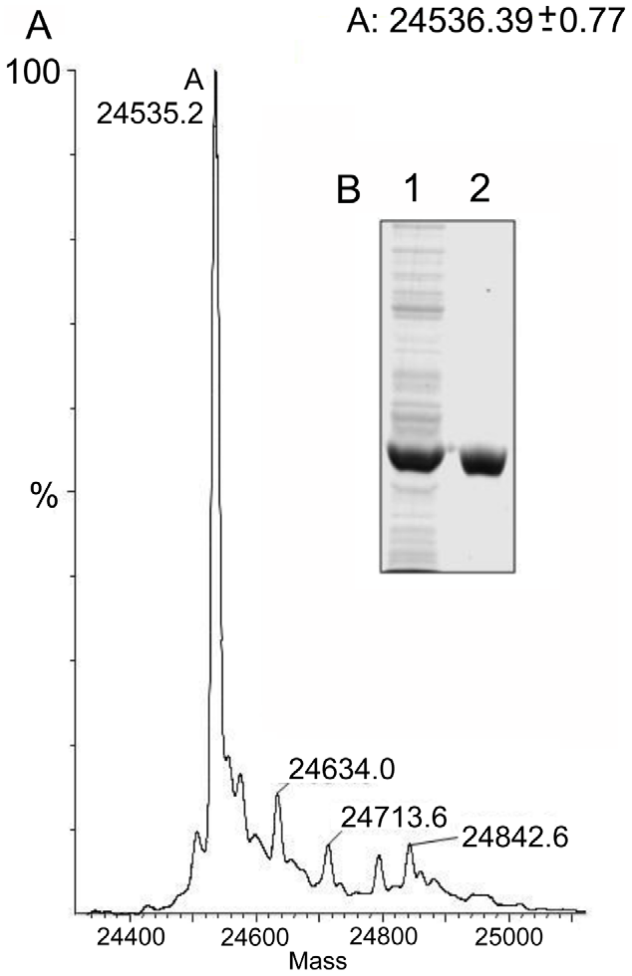
Expression and purification of recombinant FhGST-S1. A) ESI mass spectrum of the GSH-affinity purified rFhGST-S1 showing the MW of rFhGST-S1 at 24536.39±0.77 Da. B) SDS-PAGE gel of the expression and purification of rFhGST-S1. Lane 1. *E. coli* total cytosolic protein. Lane 2. GSH-affinity purified recombinant rFhGST-S1 protein. Ran on 12.5% SDS PAGE and coomassie blue stained.

Full sequence length recombinant *F. hepatica* Sigma Class GST (rFhGST-S1) was shown to be purified to a high level from transformed *E. coli* cytosol following expression yielding 57.3 mg of rFhGST-S1 from a 1 litre culture of BL21 (DE3) cells. Purity was judged by the presence of a single band upon SDS-PAGE at the estimated size and a dominating single peak via ESI MS at the precise calculated theoretical mass for the complete protein sequence (Figure 1). Analysing this fraction by 2D SDS-PAGE revealed a single protein resolving into 3 protein spots. Western blotting of the 2DE profile with anti-rFhGST-S1 antibody confirmed all 3 resolved protein spots as rFhGST-S1 (2DE and western blot data not shown). No recognition was seen probing the 3 spots with an anti-Mu class antibody.

rFhGST-S1 was produced as an active protein, displaying significant enzymic activity towards the model GST substrate 1-chloro-2,4-dinitrobenzene (CDNB) and a range of substrates commonly used to characterise GSTs (Table 1). *F. hepatica* GST is very similar in terms of its enzymatic profile to the GST of *S. japonicum* currently undergoing clinical vaccine trials. FhGST-S1 also displays higher glutathione-dependent lipid peroxidase activity compared to both Sm28GST and Sj26GST [47]. Interestingly, ligand inhibition studies on rFhGST-S1 showed the enzymic activity of rFhGST-S1 with CDNB was inhibited by the major pro-active form of the main liver fluke drug Triclabendazole. The sulphoxide derivative (TCBZ SO) gave an IC50 (50% enzyme inhibition) of 57±5 µM (5 replicates). Bile acids, potentially natural ligands for liver fluke tegumental associated proteins in the host bile environment, were also assessed for activity inhibition. The rFhGST-S1 interacted with all three bile acids tested using five replicate assays: Cholic acid (IC50 302±73 µM); Deoxycholic acid (IC50 223±21 µM) and Chenodeoxycholic acid (IC50 64±9 µM).

**Figure 2:**
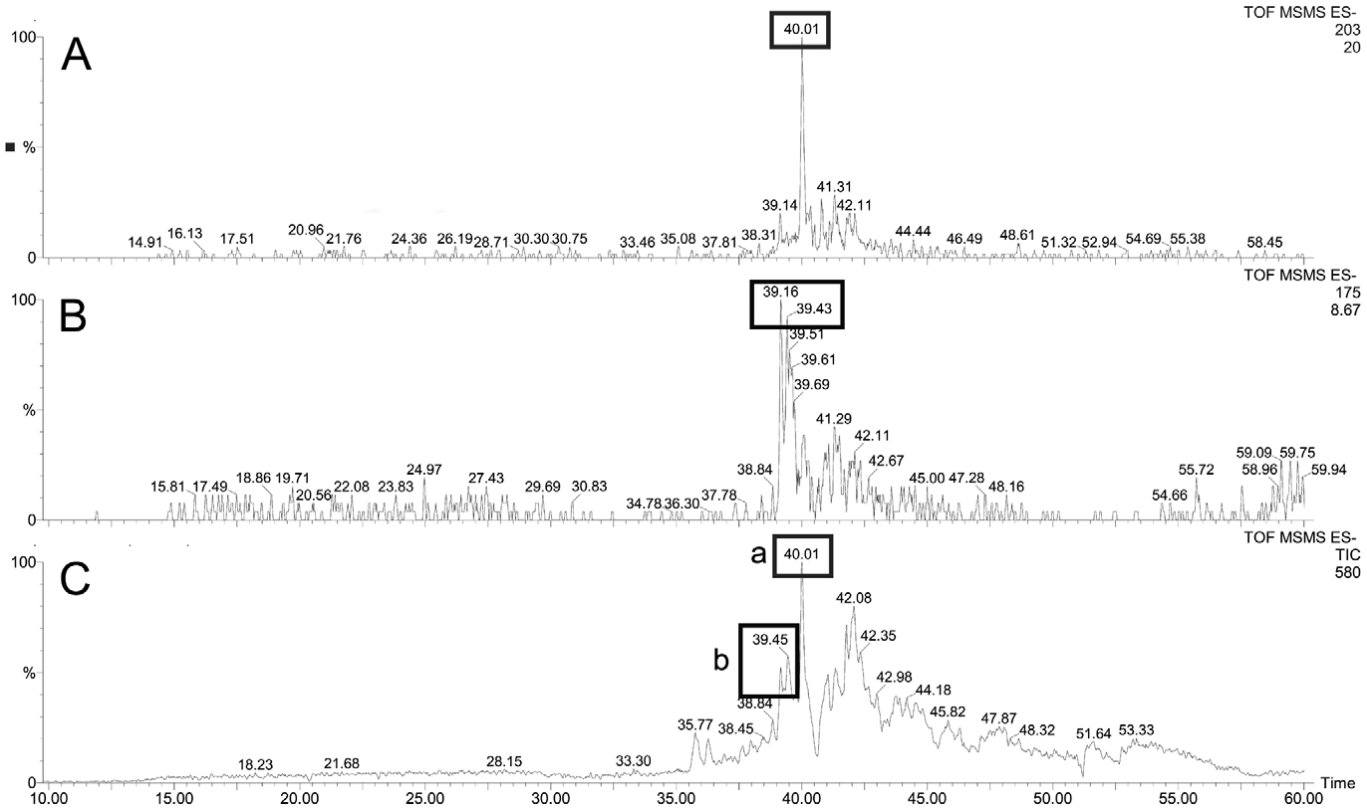
Detection of prostaglandin synthase activity of rFhGST-S1 via a mass spectrometry approach. A coupled assay with rFhGST-S1 and COX-1 catalyses the conversion of arachidonic acid to the H2 form before the prostaglandin isomer is converted to either the D or E form. Nano-LC/MS analysis allowed detection of both PGD2 (A) and PGE2 (B) in the assay mixture with the PGD2 form being the more abundant of the two prostanoids (C). Boxed figures above peaks show the fragmentation ions specific to detection of PGD2 (a) and PGE2 (b) according to the method of Schmidt *et al.* [39].

Previous studies on the Sigma class GSTs from both mammals and helminth parasites have revealed a capacity to synthesise Prostaglandin D2 (PGD2) and PGE2. Since prostaglandin synthase activity may be a conserved role of Sigma class GSTs, we also tested the ability of rFhGST-S1 to synthesise prostaglandin eicosanoids using a coupled assay with COX-1. COX-1 catalyses the conversion of arachidonic acid to the H2 form before the prostaglandin isomer is converted to either the D or E form. Nano-LC/MS analysis enabled us to detect the presence of both PGD2 and PGE2 in the assay mixture with the PGD2 form being the more abundant of the two prostanoids (Figure 2). While some PGE2 in the mixture could have arisen from rapid degradation of the unstable PGH2, nano-LC-MS was unable to detect either PGD2 or PGE2 in negative control reactions lacking either COX-1 or GST. The rFhGST-S1 catalyses PGD2 formation in a concentration-dependent manner as previously described for rOvGST-1 [26]. PGD2 was also detected in coupled assays with rFhGST-S1 and COX-1 using an Enzyme Immno Assay (EIA) detection kit (Cayman) and showed similar results (results not shown).

### Tissue localisation of Sigma GST

**Figure 3:**
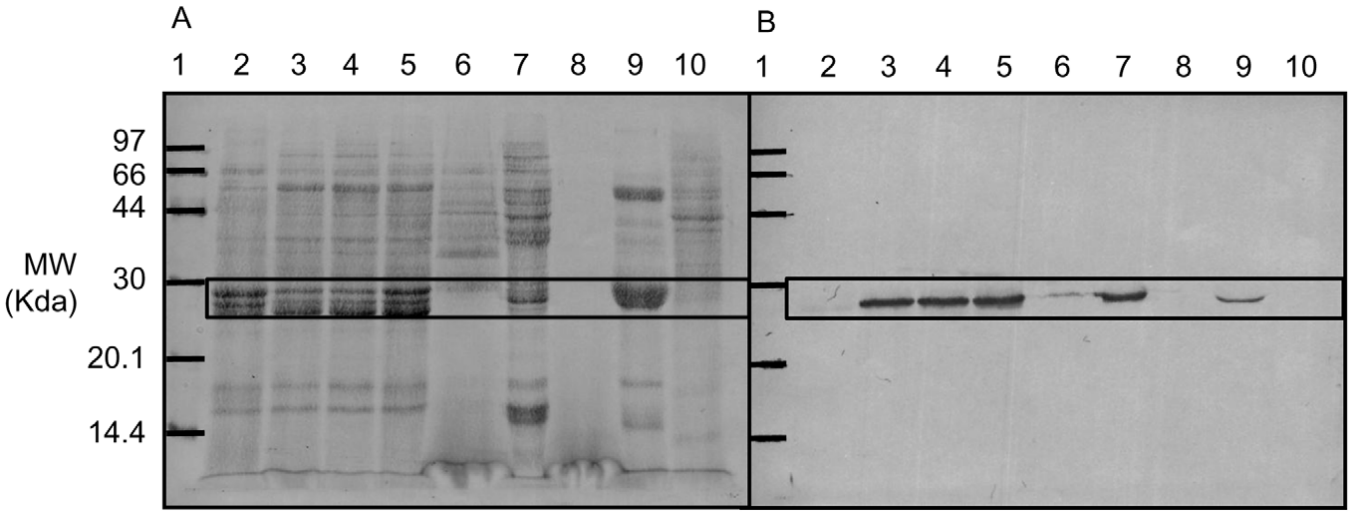
Western Blotting localising FhGST-S1 in embryonating eggs, NEJs, adults and adult ES products. 10 µg of each protein sample was resolved through 14% SDS PAGE and electrophoretically transferred to Hybond C nitrocellulose membrane. Membranes were amido black stained membrane to assess protein transfer (A) Membranes were incubated with anti-FhGST-S1 antibody diluted 1∶30,000 and developed using the BCIP/NBT liquid substrate system according to manufacturer's instructions (B). 1: Low Molecular Weight Marker (GE Biosciences); 2: Day 0 Egg; 3: Day 2 Egg; 4: Day 7 Egg; 5: Day 9 Egg; 6: NEJ Somatic Sample; 7: Adult Somatic Sample; 8: NEJ ES Products; 9: Adult ES Products; 10: Uninfected *Galba truncatula* –ve Control.

**Figure 4:**
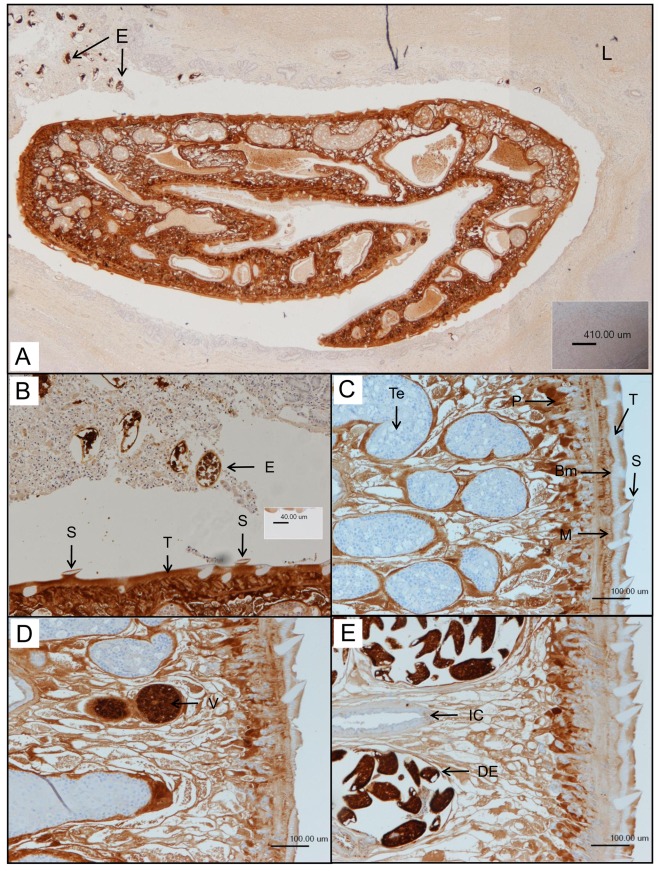
Images of FhGST-S1 localisation within *F. hepatica* tissue. A) Anti-*F. hepatica* FhGST-S1 immunohistochemical stain of a fluke in cross section within the host sheep liver bile duct. Heavily stained eggs (E) are shown released from the fluke into the bile duct in the top left-hand corner. Brown stained areas show the presence of FhGST-S1 proteins. The lack of staining in the host liver (L) highlights the specificity of the antibody. Composite picture. B) Enlarged region of A showing the intense anti-*F. hepatica* FhGST-S1staining in the voided eggs (E). The spines (S) present in the tegument (T) can be clearly distinguished by their lack of FhGST-S1 presence. C–E) Cross sections of a *F. hepatica* adult highlighting staining of FhGST-S1 in the parenchyma (P), musculature (M),the tegument (T), basal membrane (Bm) and most intensely in the vitelline cells (V) and developing eggs (DE). No staining can be seen in the tegumental spines (S), testes (T) or the intestinal caecum (IC).

FhGST-S1 was first identified in adult liver fluke in S-hexyl-GSH affinity isolated fractions of cytosol [11]. Western blots confirmed the presence of FhGST-S1 in NEJs and adult flukes and further enabled us to identify the Sigma GST in relative abundance in egg extracts, suggesting that it may play a metabolic role in embryogenesis/reproduction (Figure 3). Western blot analyses demonstrate that FhGST-S1 is consistently expressed during the course of *in vitro* parasite embryonation (days 1–9, only data for days 2, 7 and 9 shown in Figure 3). In contrast, immunoblot analysis of freshly voided (day 0) eggs reveals that expression of the Sigma class GST is greatly reduced at the time of voiding from the host (Figure 3). However, immunolocalisation studies of adult parasites revealed an abundance of FhGST-S1 in the vitelline cells and eggs, emphasising the likely importance of this enzyme in egg formation and development. Some staining was also found in the parasite parenchyma and tegument, also suggesting a role at the host-parasite interface (Figure 4). Indeed, FhGST-S1 was detected in ES products of adult fluke cultured *in vitro* (Figure 3) suggesting that the protein could, in principle, come into contact with the host immune system as it is released from the tegument during tegumental turnover and sloughing of the fluke body surface.

### Influence of rFhGST-S1 on prostaglandin synthesis in host immune cells

**Figure 5:**
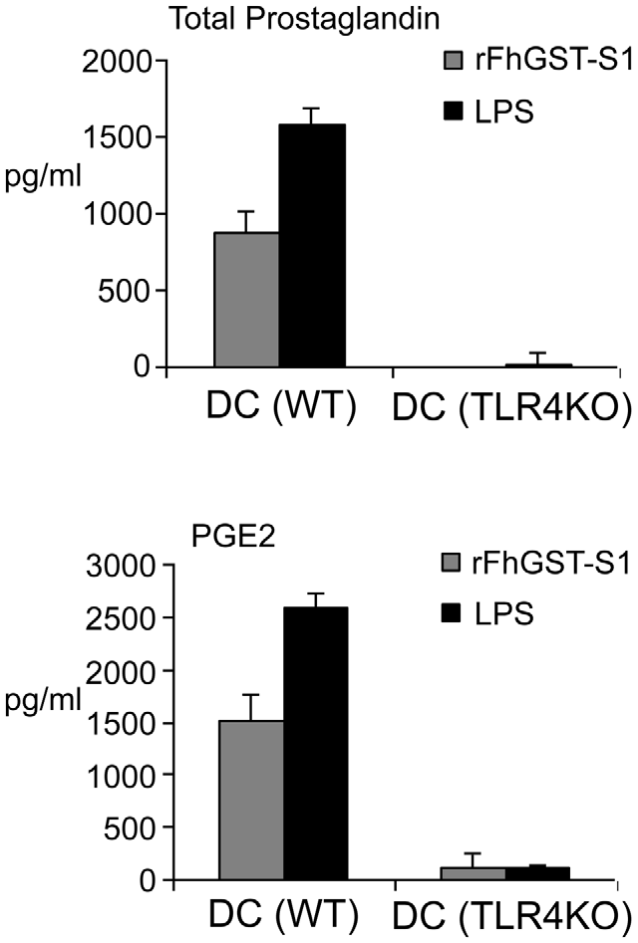
rFhGST-SI stimulates the production total prostaglandin and PGE2 from dendritic cells (DCs) in a TLR4 dependent manner. DCs derived from the bone marrow from C57BL/6j mice were cultured *in vitro* with medium, rFhGST-S1 (10 µg/ml) or LPS (100 ng/ml) for 18 hours, and the production of total prostaglandin, PGE2 and PGD2 (data for PGD2 not shown) released into supernatants determined by competitive EIA. Data are presented as the mean ± SEM following subtraction of medium controls and are representative of two experiments. WT – wild type; TLR4KO – Toll like receptor 4 knock out.

**Figure 6:**
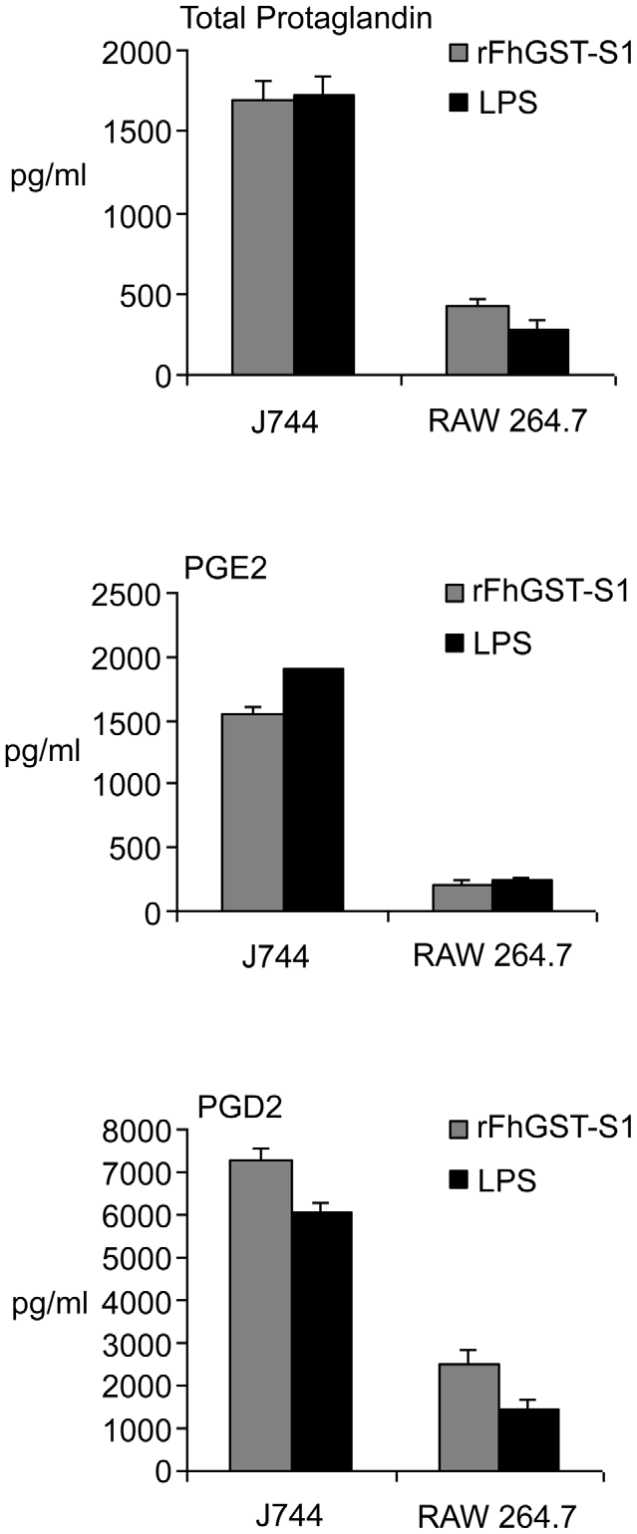
rFhGST-SI stimulates the production PGE2 and PGD2 from the macrophage cell lines J774 and RAW264.7. J744 and RAW264.7 macrophage cell lines were cultured *in vitro* with medium, rFhGST-S1 (10 µg/ml) or LPS (100 ng/ml) for 18 hours, and the production of total prostaglandin, PGE2 and PGD2 released into supernatants determined by competitive EIA. Data are presented as the mean ± SEM following subtraction of medium controls and are representative of two experiments.

rFhGST-S1 exhibited prostaglandin synthase activity producing PGE2 and PGD2. In addition, it has been shown previously that rFhGST-S1 activates DCs *in vitro* [48]. Therefore, an attempt to determine if rFhGST-S1 could induce the secretion of total prostaglandin, PGE2 and PGD2 from DCs was performed. Prior to experimentation, endotoxin levels in rFhGST-S1 were assessed and were similar to that of the media alone. Both of which were below the lower limit of detection (<0.01 EU/ml). When examining prostaglandin induction DCs stimulated with rFhGST-S1 secreted total prostaglandin and PGE2 (DC (WT); Figure 5) but not PGD2 (data not shown). Since it has been previously determined that the activation of DCs by rFhGST-S1 was dependent upon TLR4 [48] we repeated the experiment in DCs from TLR4KO mice and in keeping with previous findings demonstrated that the secretion of total prostaglandin and PGE2 by rFhGST-S1 was significantly reduced in the absence of the TLR4 receptor (DC (TLR4KO); Figure 5). rFhGST-S1 was then further assessed for its potential to induce prostaglandin secretion from macrophages by exposing two macrophage cell lines with rFhGST-S1. After 18 hours the levels of total prostaglandin, PGE2 and PGD2 were measured. In this assay, both macrophage cells lines stimulated with rFhGST-S1 secreted total prostaglandin, PGE2 and PGD2 (Figure 6). However, the levels secreted by J744 cell line were higher when compared to the amount secreted by RAW264.7 cell line. In these experiments we included medium only as a negative control and LPS as a positive control. In all experiments the levels of prostaglandin in response to rFhGST-S1 was comparable to the levels secreted in responses to LPS.

### Assessment of goat vaccinations with rFhGST-S1 challenged with *F. hepatica*

Following the completion of the vaccine trial, liver fluke were recovered and the livers scored. The resulting data is summarised in Table 2. When assessing fluke burdens, length, weight and fecal egg counts, no significant differences between rFhGST-S1 immunised and Quil A immunised groups were observed. Despite this lack of significance, at 7–9 days post-infection (dpi) the number of gross hepatic lesions appeared reduced in rFhGST-S1 immunised groups compared to the Quil A control group. At 15 weeks post-infection (wpi), a similar outcome is observed. Liver hepatic lesion scoring appeared to show reductions in the severity of damage occurred in the rFhGST-S1 immunised group compared to the Quil A only group, despite no significant differences in the aforementioned morphometric data.

**Table 2:** Results of parasitological and hepatic gross morphometric studies from vaccination.

Group	Parasitological Study			FEC (epg)		Gross Hepatic Leisons			Liver Scores (Number of Animals)				
	Fluke Burdens	Fluke Length (mm)	Fluke Weight (g)	12 WPI	13 WPI	7 DPI	8 DPI	9 DPI	− (0)	+ (<10)	++ (10–25)	+++ (25–50)	++++ (>50)
**1**	59±32.5	17.3±3	4.4±2.4	82.1	96.4	26	48	89	0	2	3	2	0
**2**	55.2±12.4	16.8±3	4.6±3.1	100	110.7	85	165	172	0	1	2	3	0

Group 1 (goats immunised with recombinant FhGST-S1) and Group 2 (infected control group immunised with Quil A only). Liver scores were recorded at necropsy 15 wpi. WPI – Weeks post infection. DPI – Days post infection.

**Figure 7:**
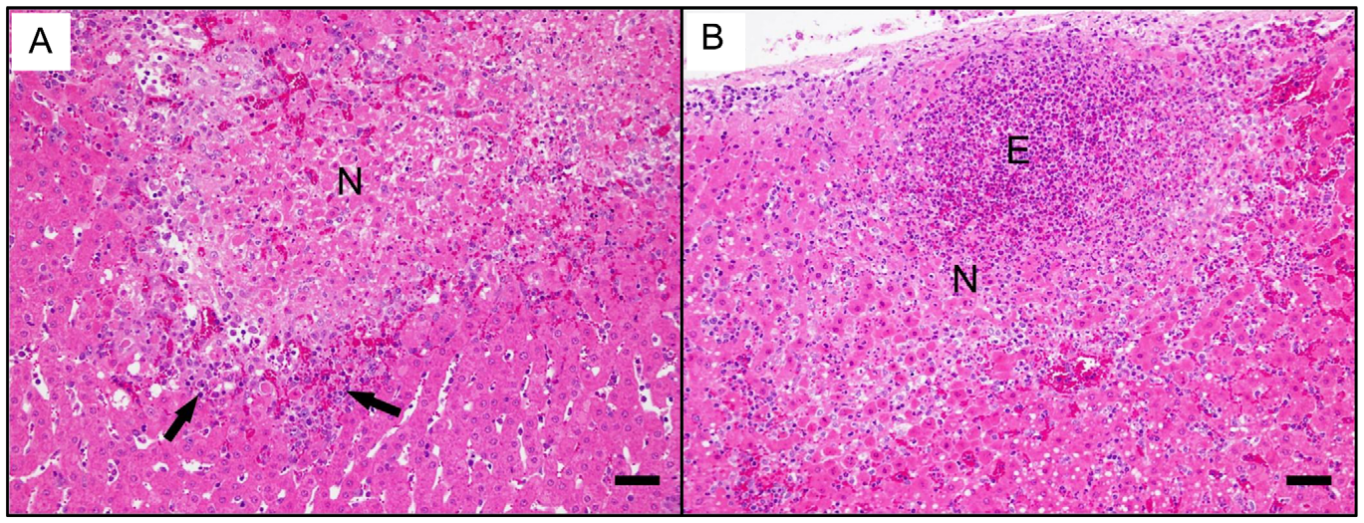
The effects of vaccination with rFhGST-S1 or Quil A. A) Photomicrograph of the liver from the Quil A immunised group showing an area of coagulative necrosis (N) surrounded by scarce inflammatory infiltration (arrows) with occasional eosinophils. B) Photomicrograph of the liver from the rFhGST-S1 immunised group showing a coagulative necrotic area (N) associated to numerous eosinophils (E). Both images haematoxylin and eosin stained. Both bar represent 100 µm.

Microscopically, at 7–9 dpi animals from the Quil A group showed tortuous necrotic tracts surrounded by a scarce inflammatory infiltration with occasional eosinophils (Figure 7A). Older necrotic areas were surrounded by macrophages, epithelioid cells and multinucleate giant cells and lymphocytes. Some migrating larvae were found in the liver parenchyma without inflammatory infiltrate associated to them. In goats immunised with rFhGST-S1 smaller necrotic areas associated to a heavy infiltration of eosinophils (Figure 7B) were seen. Unlike the Quil A immunised group, all migrating larvae found were surrounded by a heavy infiltration of eosinophils.

**Figure 8:**
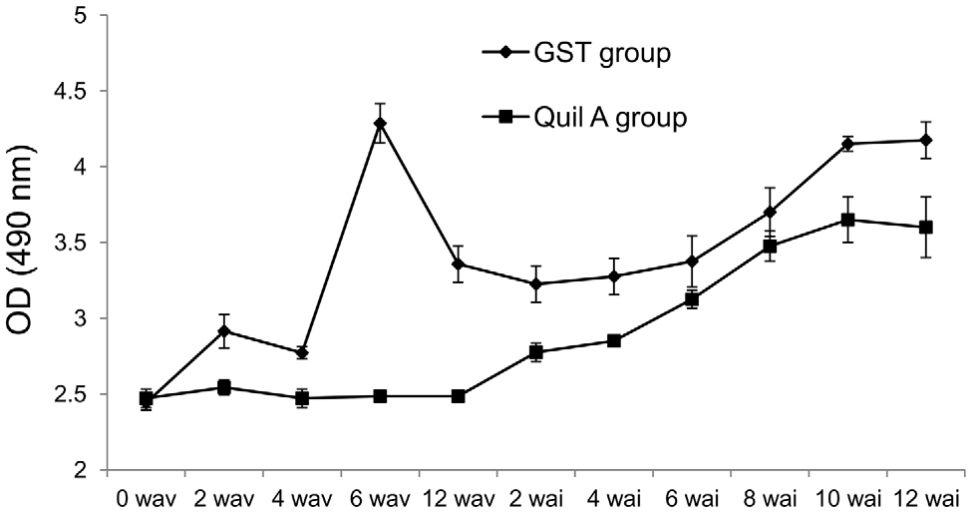
Specific IgG response. Serum titres of IgG anti-r-FhGST-S1 at 0, 2, 4 and 6 weeks after vaccination (wav) and at 2, 4, 6, 8, 10 and 12 weeks after infection (wai). Results expressed in log_10_.

A significant increase of IgG anti-rFhGST-S1 was observed two weeks after vaccination with a strong increase after the second injection at week 4 in immunised animals (Figure 8). The Quil A control group did not show any specific IgG response until 2 weeks after infection. Specific IgG titres increased during infection in both groups, but they were consistently higher in the immunised group throughout the duration of the the experiment.

## Discussion

Previous studies have highlighted the importance of parasite GSTs, including Sigma class GSTs, in host-parasite interactions and as potential vaccination candidates. With this in mind, we have studied the relatively newly identified Sigma class GST from *F. hepatica* to both enhance our understanding of this important enzyme in *Fasciola* and the Sigma class of GSTs as a whole.

Alignments and phylogenetics classified FhGST-S1 alongside trematode and mammalian Sigma class GSTs, yet there remains a distinct divide between the parasites and their hosts, a phenomenon also observed for the recently reclassified ‘Nu’ class of GSTs from nematodes [49]. Therefore, it may be that trematode GSTs are sufficiently distinct to support a sub-classification within the broad Sigma class. The distinction of FhGST-S1 from fasciolosis host Sigma class GSTs enhances its potential as a therapeutic target.

Substrate activity profiling of rFhGST-S1 using model substrates showed the enzyme to have comparable activity to other trematode Sigma class GSTs such as Sm28GST [47]. However, rFhGST-S1 exhibits relatively high GSH-conjugating activity towards the potentially natural reactive aldehyde, 4-hydroxy-nonenal (4-HNE) toxin and high GSH-dependent peroxidase activity towards the tested lipid peroxides which includes the endogenous substrate linoleic acid hydroperoxide. 4-HNE is the major aldehydic end-product of lipid peroxidation that is involved in signalling of host immune cells leading to apoptosis of T- and B-cells [50].

Assessing the inhibition of rFhGST-S1 activity with CDNB revealed that both bile acids and the flukicide TCBZ appear to bind to the enzyme. In particular, the interaction of the bile acid cholate with rFhGST-S1 is approximately ten fold higher than GSTs from the sheep intestinal cestode *Moniezia expansa* [51]. Host bile acids are known as triggers of physiological processes in trematodes including *Fasciola* sp. [52], [53]. Therefore, molecular interaction of bile acids with FhGST-S1 warrants further investigation especially, given that FhGST-S1 is localised to near the body surface of the fluke, where it could potentially bind cholate and other free bile acids found in abundance in host bile (cholate is found at approximately 100 mM in sheep bile) [54]. The hydroxy-TCBZ SO levels in the bile have been shown to be in excess of 100 µM [55] thus, the IC50 of 57±5 µM for TCBZ SO suggests the abundant FhGST-S1 could be involved in TCBZ response in phase III sequestration based detoxification. This finding warrants further investigation to understand the role of FhGST-S1 in TCBZ action or detoxification.

Sigma class GSTs from both parasites and mammals have been known to exhibit prostaglandin synthase activity. To this end, the Sigma GST from *F. hepatica* shares a high sequence identity with recognised Sigma class GSTs with prostaglandin synthase activity, including rOvGST-1 from the filarial parasite, *Onchocerca volvulus*. Using a coupled assay with COX-1 we have shown that rFhGST-S1 is capable of synthesizing both PGD_2_ and PGE_2_, with PGD_2_ being the predominant prostanoid. Parasite-derived eicosanoids, including prostaglandins, are known to be important in the establishment of parasitic infection and the survival and proliferation within the host. Therefore, eicosanoids produced by parasitic helminths may play a role in pathophysiological changes during helminth infections. For example, chronic fasciolosis is associated with fever and changes in liver biochemistry, both of which could be associated with parasite-derived eicosanoids thromboxane B2 (TXB2), PGI_2_, PGE_2_ and leukotriene B4 (LTB_4_), detected in the ES products and homogenates of adult *F. hepatica* worms [56]. In addition, the migration of host epidermal Langerhans cells, which play a key part in immune defence mechanisms, has been shown to be inhibited by parasite-derived PGD_2_ in the *Schistosoma mansoni*-mouse model of human infection, thus allowing schistosomes to manipulate the host immune system [57]. Earlier studies have revealed the presence of eicosanoids produced by *S. mansoni* cercariae which could also play a role in establishment of infections through loss of the cercarial tail following penetration of the skin [58]. It therefore seems likely that prostaglandins synthesised via FhGST-S1 will have a role in establishing the infection within the host.

In general, prostaglandins and eicosanoids have potent biological activities in reproduction. For example in the zebrafish egg, high levels of PGE_2_ were seen post fertilisation coupled with high PGD_2_ synthase transcript levels during the early stages of egg development concomitant with an exponential decrease of PGD_2_ levels over the next 120 h post fertilisation [59]. However, in *F. hepatica*, eggs in gravid adults are released in an immature state in the bile duct, where they pass to the external environment via the host's excretory system and complete embryogenesis ex-host. Therefore, FhGST-S1 may have a secondary, or indeed primary, function in egg development and embyrogenesis. A role in egg development is further supported by proteomic studies of *F. hepatica* ontogenic stages which reveal the presence of FhGST-S1 in eggs ([42] and the current study).

FhGST-S1 appears to be highly abundant in eggs with western blotting showing FhGST-S1 to be constitutively expressed, despite its association with a large spot consisting of multiple co-migrating proteins unresolved via 2DE (for association see [42]). Immunolocalisation studies revealed that FhGST-S1 is closely associated with vitelline cells of mature adult worms. Given the importance of PGs in reproduction, we hypothesize that PG synthase activity exhibited by rFhGST-S1 contributes to developmental cues during egg formation. Interestingly, no FhGST-S1 was seen in day 0, un-embryonated, eggs by western blotting yet *in situ* immunlocalisation showed freshly voided eggs, equivalent to day 0 eggs, to contain copious amounts of FhGST-S1. While it is most likely that FhGST-S1 is present in day 0 eggs, albeit at a reduced expression, the discrepancy seen between the two techniques is probably related to the antibody dilutions used for each method; in total a 40-fold difference in favour of immunolocalisation.

FhGST-S1 was also identified in both NEJs and adult worms using western blotting. This finding emphasises the multi-functionality of FhGST-S1, where in NEJs egg productions is not yet in process, suggesting its main function is in PG synthesis for host modulation or as a detoxification enzyme. In the adult worm, FhGST-S1 could also be localised, to a smaller extent, in the parenchyma and tegument. Given the high activity of FhGST-S1 towards the toxic 4-HNE and to lipid hydroperoxides this suggests a detoxification role at the host-parasite interface.

With near surface expression of FhGST-S1, in the parenchyma and tegument, there is the potential for this enzyme to be readily released into the host environment. Indeed, we have identified FhGST-S1 in the ES products of adult worms. With this in mind, previous studies have highlighted the importance of parasite Sigma class GSTs in immunomodulation of the host immune response. This includes our recent study implicating rFhGST-S1 in chronic inflammation through the activation of dendritic cells (DCs) [48]. While active rFhGST-S1 was able to induce levels of IL-12p40 and IL-6 cytokines in DCs in a dose-dependent manner, the previously described *F. hepatica* Mu-class GSTs failed to induce any cytokine secretion. Since denatured rFhGST-S1 also failed to induce any cytokines in DCs, activation of DCs is likely related to the structure and activity of the enzyme. However, inhibition of nitric oxide production, involved in driving a Th2 immune response, may also be a contributing factor in skewing the host response to fasciolosis [60].

*F. hepatica* infections are associated with a T-helper-cell type 2 (Th2) immune response dominating during the chronic phases of infection [61], but pro-inflammatory responses are suppressed [62]. Suppression of allergic responses during chronic parasitic worm infections has a mutually beneficial effect on the parasites' proliferation and the hosts' survival. Prostanoids, including PGD_2_, are important in mediating these allergic inflammatory responses. While generally regarded as pro-inflammatory molecules, these important lipid molecules are also involved in mediating anti-inflammatory responses [63]. Helminth-derived molecules are thought to be involved in driving the Th2 response stereotypical of parasitic worm infections. DC and macrophage cell cultures exposed to rFhGST-S1 showed elevated levels of Th2 cytokines after 24 h [48]. In this study, the effects of rFhGST-S1 exposure onprostanoid synthesis in host immune cells was investigated. The results of which show the stimulation of PGD_2_ and PGE_2_ in both DCs and macrophage cell lines suggesting FhGST-S1 is one such helminth derived molecule capable of driving the Th2 response.

As we have shown FhGST-S1 to have key roles in *F. hepatica*, both in NEJs and adult worms, coupled with the near surface expression and release of the enzyme via the ES products, we assessed the potential of FhGST-S1 to be used as a vaccine candidate. This was especially poignant given that the *S. mansoni* Sigma GST homologue (Sm28) is in phase II clinical trials [12]. Unfortunately, the current goat based vaccine trial did not show any significant differences in fluke burdens between the rFhGST-S1 immunised and Quil A control group. However, a high individual variability was recorded, particularly in the vaccinated group also reported in previous trials using goats vaccinated with alternative candidates such as cathepsin L1 [64] and Sm14 [65]. The vaccine trial shown here using a target species with an acceptable adjuvant may have been adversely affected by the strain of *F. hepatica* used to challenge goats. Here we have shown an unusually high infectivity rate with the strain of *F. hepatica* used; which we have reported in a previous trial using goats [64]. Using an alternative strain of *F. hepatica* for experimental infections in this species has given normal infectivity rates ranging from 14% to 26.5% [65].

In the present trial it appeared that goats immunised with rFhGST-S1, despite no variations in fluke burdens or morphometrics, showed reduced gross hepatic lesions during early infection, up to day 9 post infection, which continued to week 15 post infection where liver scores for hepatic lesions appeared reduced for rFhGST-S1 immunised animals. These results suggest that animals from the immunised group produced an early response to migrating larvae that has induced some partial protection from liver damage. The early and consistent specific IgG response found in the present work also agrees with the results obtained in a previous trial using naïve FhGST [46]. However, in both studies high levels of specific IgG did not induced a protective response reducing worm burdens.

A promising aspect of producing anti-helminth vaccines is developing multivalent vaccines. In many cases the greatest protection from challenge is by vaccinating with a combination of *Fasciola* antigens [66], [67]. Therefore, based on the immunisation with FhGST-S1 showing an early response reducing hepatica damage, could be considered for inclusion into a multivalent vaccine against Fasciolosis. In addition, in light of our findings showing FhGST-S1 to be highly prominent in egg production and the egg itself, as with previous vaccination trials [67], it will be important to investigate the ability of eggs voided from vaccinated animals to embryonate. The potential to reduce pasture contamination by inhibiting egg embryonation, combined with the demonstrated reduction in liver damage, warrants further exploration using rFhGST-S1 as a vaccine candidate.

In summary, we have further promoted the concept that FhGST-S1 clearly demonstrates key host-parasite roles in synthesising PGs and stimulating PG release from host innate immune cells. In addition we have shown FhGST-S1 to be a key protein for detoxification, which may well be involved in TCBZ response. In line with current vaccine development theory we have shown FhGST-S1 to have multi-functional roles in the liver fluke physiology. Furthermore, we have shown FhGST-S1 to be expressed across ontogenic stages, localised to the fluke surface, and to the egg, both characteristics vital for vaccine development and success. Whilst no protection from fluke burden was seen in trials, the inclusion of rFhGST-S1 as a multivalent vaccine component should be investigated. However, it is important to fully characterise the host immune response during the early stages post-infection to better understand the mechanism mediating an effective host response. This will be essential to improve any future vaccine formulation.

[36,51,68–71] Table 2 Refs.

## Supporting Information

Figure S1 **Multiple sequence alignment and neighbour-joining phylogenetic tree across seven species-independent classes of GSTs.** A) Alignment of the sigma class GSTs of trematodes shows the extent of identity and similarity across this class of GSTs. Boxed residues indicate complete identity between all sequences. Residues shaded in grey indicate conserved residues. B) Neighbour-joining tree placing mammalian and trematode GSTs within the same broad Sigma class. A distinct separation of clusters within this Sigma class is observed as with the recently reclassified ‘Nu’ class of GSTs from nematodes [49]. Sequences were aligned via the ClustalW program [29] in BioEdit Sequence Alignment Editor version 7.0.5.2. [30]. Phylogenetic neighbour-joining bootstrap trees were produced and viewed within TREEVIEW [33]. Key to sequences in 1a and 1b. *Xenopus laevis*; Fhep49c06_omega *Fasciola hepatica*; Fhep54b04_omega *Fasciola hepatica*; O09131_GSTO1_MUS *Mus musculus*; O35543_PTGD2lowbar;RAT *Rattus norvegicus*; O60760_PTGD2_HOMO *Homo sapiens*; O73888_PTGD2_GGAL *Gallus gallus*; O97096_GST_CLOSI *Clonorchis sinensis*; P08263_GSTA1_HOMO *Homo sapiens*; P08515_GSTM_SCHJA *Schistosoma japonicum*; P09488_GSTM1.1_HOMO *Homo sapiens*; P09792_GST28_SCHMA *Schistosoma mansoni*; P10299_GSTP1_CE *Caenorhabditis elegans*; P19157_GSTP1_MUS *Mus musculus*; P20432_GSTT1_DROME *Drosophila melanogaster*; P28801_GSTP1_BOVIN *Bos taurus*; P30113_GST28_SCHBO *Schistosoma bovis*; P30114_GST28_SCHHA *Schistosoma haematobium*; P34345_GSTO_CE *Caenorhabditis elegans*; P35661_GSTM_SCHMA *Schistosoma mansoni*; P41043_GSTS1_DROME *Drosophila melanogaster*; P46436_GSTS1_ASCSU *Ascaris suum*; P51781_GSTA1_PIG *Sus scrofa*; P78417_GSTO1_HOMO *Homo sapiens*; P80031_GSTP1_PIG *Sus scrofa*; P91253_GSTS7_CE *Caenorhabditis elegans*; Q000H8_GSTM2_PIG *Sus scrofa*; Q06A71_FhGST-S1 *Fasciola hepatica*; Q26200_GST_PARWE *Paragonimus westermani*; Q26513_GST_SCHJA *Schistosoma japonicum*; Q28035_GSTA1_BOVIN *Bos taurus*; Q30B87_GSTM3_SHEEP *Ovis aries*; Q58ET5_GSTM1_MUS *Mus musculus*; Q5TZY2_GSTT1_HOMO *Homo sapiens*; Q5TZY3_GSTP1_HOMO *Homo sapiens*; Q6IB17_GSTZ1.1_HOMO *Homo sapiens*; Q6P8Q0_GSTA1_MUS *Mus musculus*; Q86LC0_GSTO_SCHMA *Schistosoma mansoni*; Q8ISK1_GST_OPIVI *Opisthorchis viverrini*; Q91X50_GSTT1_MUS *Mus musculus*; Q9JHF7_PTGD2_MUS *Mus musculus*; Q9N0V4_GSTM1_BOVIN *Bos taurus*; Q9N2J6_GSTMic_SHEEP *Ovis aries*; Q9N4H6_GSTZ43_CE *Caenorhabditis elegans*; Q9NAW7_GSTS_HCON *Haemonchus contortus*; Q9TTY8_GSTP1_CAPHI *Capra hircus*; Q9WVL0_GSTZ1_MUS *Mus musculus*; Q9XS30_GST_SHEEP *Ovis aries*; XP_535659.1_PGDS_CFAM *Canis familiaris*. A1BNE5_GST_CLOSI *Clonorchis sinensis*; AAH53774.1_GSTS1-1_XLAE
(TIF)

Table S1 **Amino acid identity comparisons of FhGST-S1 with GSTs from cytosolic classes across a variety of taxa.** Amino acid sequence comparison of FhGST-S1 with other trematode GSTs clearly places FhGST-S1 into the Sigma class of GSTs, with identities averaging approximately 45%. Comparison with the most closely matching mammalian GSTs shows sequence identities averaging only approximately 28%. PTGD – Prostaglandin D synthase; Mic -Microsomal.
(XLS)
