# Supplementary figures and images for: The Sigma Class Glutathione Transferase from the Liver Fluke Fasciola hepatica
Source: PLoS Negl Trop Dis. 2012 May 29;6(5):e1666. doi: 10.1371/journal.pntd.0001666 (PMC3362645; doi:10.1371/journal.pntd.0001666)

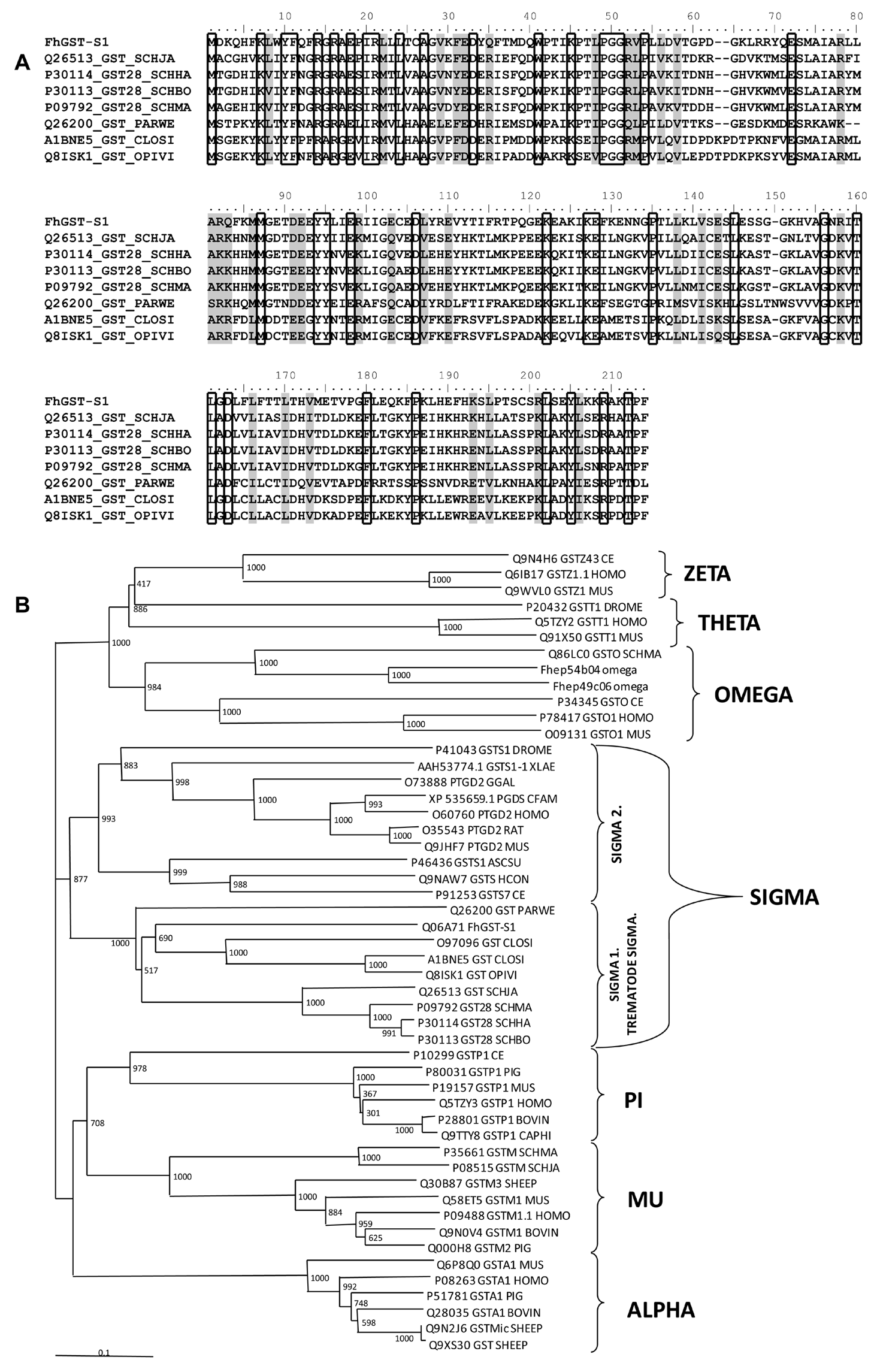

Supplement: Figure S1 — Multiple sequence alignment and neighbour-joining phylogenetic tree across seven species-independent classes of GSTs. A) Alignment of the sigma class GSTs of trematodes shows the extent of identity and similarity across this class of GSTs. Boxed residues indicate complete identity between all sequences. Residues shaded in grey indicate conserved residues. B) Neighbour-joining tree placing mammalian and trematode GSTs within the same broad Sigma class. A distinct separation of clusters within this Sigma class is observed as with the recently reclassified ‘Nu’ class of GSTs from nematodes [49]. Sequences were aligned via the ClustalW program [29] in BioEdit Sequence Alignment Editor version 7.0.5.2. [30]. Phylogenetic neighbour-joining bootstrap trees were produced and viewed within TREEVIEW [33]. Key to sequences in 1a and 1b. Xenopus laevis; Fhep49c06_omega Fasciola hepatica; Fhep54b04_omega Fasciola hepatica; O09131_GSTO1_MUS Mus musculus; O35543_PTGD2lowbar;RAT Rattus norvegicus; O60760_PTGD2_HOMO Homo sapiens; O73888_PTGD2_GGAL Gallus gallus; O97096_GST_CLOSI Clonorchis sinensis; P08263_GSTA1_HOMO Homo sapiens; P08515_GSTM_SCHJA Schistosoma japonicum; P09488_GSTM1.1_HOMO Homo sapiens; P09792_GST28_SCHMA Schistosoma mansoni; P10299_GSTP1_CE Caenorhabditis elegans; P19157_GSTP1_MUS Mus musculus; P20432_GSTT1_DROME Drosophila melanogaster; P28801_GSTP1_BOVIN Bos taurus; P30113_GST28_SCHBO Schistosoma bovis; P30114_GST28_SCHHA Schistosoma haematobium; P34345_GSTO_CE Caenorhabditis elegans; P35661_GSTM_SCHMA Schistosoma mansoni; P41043_GSTS1_DROME Drosophila melanogaster; P46436_GSTS1_ASCSU Ascaris suum; P51781_GSTA1_PIG Sus scrofa; P78417_GSTO1_HOMO Homo sapiens; P80031_GSTP1_PIG Sus scrofa; P91253_GSTS7_CE Caenorhabditis elegans; Q000H8_GSTM2_PIG Sus scrofa; Q06A71_FhGST-S1 Fasciola hepatica; Q26200_GST_PARWE Paragonimus westermani; Q26513_GST_SCHJA Schistosoma japonicum; Q28035_GSTA1_BOVIN Bos taurus; Q30B87_GSTM3_SHEEP Ovis aries; Q58ET5_GSTM1_MUS Mus musculus; Q5 [file pntd.0001666.s001.tif]
